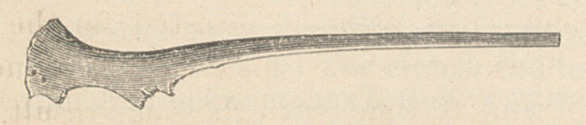# An Irreducible Hernia Containing a Bone

**Published:** 1873-10-15

**Authors:** A. C. Olney

**Affiliations:** Chillicothe, Iowa


### AN IRREDUCIBLE HERNIA CONTAINING A BONE.

CASE REPORTED TO THE WAPELLO COUNTY, IOWA, MEDICAL SOCIETY, BY A. C. OLNEY, M.D., CHILLICOTHE, IOWA.

July, 20th, 1873, 11 o'clock, A.M., in company with Dr. Hyatt, of Chillicothe, visited S. C—, a farmer, aged 75 years, suffering from an irreducible oblique inguinal hernia of the right side.

The history of the case is as follows, as we learned it from himself and his family: He had been troubled with this hernia about eight years, and up to Thursday, the 17th of July, had experienced but little inconvenience in reducing it. On that day, he had been working in the harvest field, and on going to a well of water near by he drank freely, whereupon he immediately discovered that a portion of the bowel had come down, and that the hernial sac was larger than common; and all efforts on the part of the old gentleman to reduce it, failed; and the ordinary manipulations in reducing the hernia producing great pain, he desisted for the time. He however resumed his efforts at reduction, from time to time, with the same result, until these attempts became so painful that he was obliged to desist altogether.

The second day after this trouble commenced, he took some cathartic pills, and becoming very sick, vomitted. From this time forward, three or four times in the twentyfour hours, he was troubled with stercoraceous vomiting.

Dr. Hyatt first saw him on Saturday, the 19th, about 7 o'clock, P. M. At this time the parts about the hernial sac were inflamed, swollen, and tender to the touch. The Doctor made no attempt to reduce the hernia at this time; having applied cloths wet in cold water to the inflamed parts, and administering a dose of morphine, he left him for the night. As said before, we saw him together on the morning of the 20th. At this time the patient was placed on a lounge, and put under the influence of chloroform. Dr. Hyatt then examined the hernial sac, and discovered something unusual in its contents, and also made gentle attemps to reduce the hernia by the taxis. The Doctor directed my attention to this foreign body, which I could distinctly feel above Poupart's ligament, and near the femoral ring. And here, it is worthy of remark, was the most protuberant part of the inflamed surface, making it appear like an ordinary *femoral hernia*. For considerations I will not now mention, we concluded not to operate, and let the patient come out from the influence of chloroform. We ordered an enema of warm water, prescribed a few powders of morphine, and then left him.

July (Monday) 21st, 6 o'clock, A.M., the patient *died*. Same day, 4 o'clock, P. M., ten hours after death, assisted by Dr. Hyatt, made a post mortem examination. Rigor mortis well marked. Made an incision through the integuments over the most prominent part of the tumor, parallel with the median line, about three inches in length. Then dissecting down carefully upon Poupart's ligament, and in the direction of the femoral ring, we came in contact with the smaller end of the bone here represented (full size). Pushing aside the integuments, the superficial fascia, intercolumnar fascia, cremaster muscle, and transversalis fascia (these being agglutinated together by inflammation), exposed the peritoneal sac. About one half of the smaller end of this bone was protuding from the sac. The bone was lying above Poupart's ligament, and pointing in the direction of the femoral ring. We then cut into the peritoneal sac, disengaged the bone from the bowel, which was then readily reduced. The bowel was not strangulated, but rendered irreducible by being pierced or transfixed, and held in position by the bone. There was considerable peritonitis; the bowel was but little inflamed. But in the neighborhood of the femoral ring, to the right of the hernial sac, were considerable inflammation, serous infiltration, discoloration of the skin, and swelling.

In addition to the report of the case as above given, we will offer a few remarks:

1st. At the time this accident happened, and for some time previous, this old man had enjoyed good health for one of his age, and would perhaps have lived some years longer, had not this accident occurred.

2d. Mr. C. had been in the habit of wearing a truss, but on the morning of the 17th had neglected to put it on, as was his custom, hence the accident, which we have just narrated, verifying the precept that no one, who has a hernia, however small and inconsiderable, is safe, without wearing a suitable truss.

3d. Had the patient been seen by a careful and experienced surgeon early, before the bone had transfixed the bowel and peritoneal sac, and the surgeon had correctly diagnosed the case, he might have operated successfully by reducing the bowel, with the bone retained, and thus the patient's life might have been saved; but any time after the bone had pierced, the intestine and hernial sac, an operation would not have promised any prospect of success.

4th. Under the head of "Obstructed Irreducible Hernia," Samuel D. Gross, in his *Principles and Practice of Surgery*, Vol. II., page 521, mentions the accumulation and impaction of fecal matter and flatus as the cause. But our case does not come under this head exactly, nor are we aware that any similar case has been reported. [We do not, however, here in a country village, have access to extensive libraries.] Our case is a strange and anomalous one, and, as in all cases of difficulty, our practice should be governed by circumstances.

With regard to the duties of the surgeon in cases of irreducible and strangulated hernia, we will refer to Erichsen's, and Gross' *Principles and Practice of Surgery;* and also to Clinical Lectures on Strangulated Hernia, delivered at St. Bartholomew's Hospital, England, and published in the *Medical News and Library*, for September, October, November and December, 1872.

## Figures and Tables

**Figure f1:**